# Transactions of the American Medical Association

**Published:** 1887-04

**Authors:** 


### BOOK REVIEWS.

TRANSACTIONS OF THE AMERICAN SURGICAL ASSOCIATION. *Edited by* J. EWING MEARS, M.D. *Philadelphia: printed for the Association, and for sale by*, P. BLAKISTON, SON & COMPANY. *Volume the Third, large octavo, pp. xxxv. 395. 1885. Volume the Fourth, large octavo, pp. xxiii. 339. 1886.*

These two handsome volumes form a valuable contribution to the literature of general surgery.

The first paper of volume one, on "Operative Surgery of the Human Brain," by John B. Roberts, M.D., has been reprinted, a brief notice of which appeared in this JOURNAL AND EXAMINER lii. p. 576. The next two papers, one by Samuel W. Gross, M.D., on "Nephrætomy," collates 233 cases, from public and private sources. The various conditions for which the operation is made are given, with the absolute and relative mortality. The second, by L. McLane Tiffany, M.D., gives the particulars of an interesting case of "Nephrolithotomy" by transverse lumbar incision. The short paper of J. Collins Warren, M.D., has been expanded into the volume entitled: "The Healing of Arteries after Ligature, in Man and Animals," recently issued by Messrs. William Wood & Company, and reviewed in volume liii., page 81, of this journal. The most extensive paper by N. Senn, on "Air Embolism," has already been noticed in our columns. It represents a class of work of which there is too little attempted in this country. The remaining papers relate to the cultivation of surgical bacteria, the surgery of the hypertrophied prostate, traumatic tetanus, phosphorous necrosis of the jaws, and a device for atmospheric purification. The interesting case of cholecystotomy reported by C. T. Parkes, M.D., is among the most brilliant and difficult triumphs of abdominal surgery.

The discussions are clear and concise, and add greatly to the interest of the papers.

Volume four, though some sixty pages smaller than the foregoing, is not less interesting and valuable in its contents. The more important papers are those on "The Union of Nerves," by Moses Gunn, M.D., an exceedingly interesting paper on "Diagnostitial Laparatomy," by Christopher Johnston, M.D., followed by a discussion in which the worst points in this surgical procedure are well handled. The chief paper is by N. Senn, M.D., on the "Surgery of the Pancreas." It is perhaps not too great praise to say that both in thoroughness of treatment and accurate reasoning, this paper will compare favorably with any work of a similar scope and character ever performed in this country. The paper contains an excellent resume of the meagre literature regarding the pancreas and its diseases. In addition, the author has gathered a wealth of clinical and experimental data, which are carefully incorporated in such a manner as to place our knowledge of the surgical relations of the pancreas on a sure foundation.

Dr. Prince has added a stove-pipe to his device for atmospheric purification, and the cut of last year (plus the pipe), appears among his remarks in the discussion of Everest's paper on the "Bacteria of Surgical Diseases." A weather-vane might be added to the structure before it reappears next year.

The editor and the publishers are to be congratulated on the elegant typographical appearance of these two volumes.

